# Low-Velocity Impact Behaviour of Titanium-Based Carbon-Fibre/Epoxy Laminate

**DOI:** 10.3390/ma17215380

**Published:** 2024-11-04

**Authors:** Jing Sun, Weilin Chen, Hongjie Luo, Xingfang Xie, Jingzhou Zhang, Chao Ding

**Affiliations:** 1 School of Civil Engineering and Transportation, Guangzhou University, Guangzhou 510006, China; jingslinks@gzhu.edu.cn (J.S.); 2112416100@e.gzhu.edu.cn (W.C.); 32116160010@e.gzhu.edu.cn (H.L.); 2016160122@e.gzhu.edu.cn (X.X.); 2 Foshan Huaxun Intelligent Technology Co., Ltd., Foshan 528200, China

**Keywords:** fibre metal laminate, titanium, carbon-fibre, low-velocity impact, damage mechanism, energy absorption

## Abstract

This study investigated the low-velocity impact response of titanium-based carbon-fibre/epoxy laminate (TI-CF FML). A comprehensive experimental study was carried out with impact energies ranging from 16.9 J to 91.9 J. Finite element analysis, performed using ABAQUS, was employed to elucidate the failure mechanisms of the laminate. Three distinct damage modes were identified based on the impact energy levels. The energy absorption characteristics of the TI-CF FML were analysed, revealing that maximum energy absorption is achieved and remains constant after penetration occurs. The relationship between impact force and displacement was also explored, showing that the laminate can withstand a peak force of 13.1 kN. The research on the impact resistance, damage mechanisms and energy absorption capacity of TI-CF FML provides an in-depth understanding of the impact behaviour of the laminate and its suitability for various industrial applications.

## 1. Introduction

One of the promising materials considered for the next generation of fibre metal laminate (FML) is the titanium-based carbon-fibre/epoxy laminate (TI-CF FML) [1,2,3,4]. This laminate consists of carbon-fibre/epoxy (CF/E) composite embellished with a thin titanium layer featuring excellent fatigue resistance, corrosion resistance and fire resistance [5,6,7,8,9], making it an ideal material for applications in aerospace, automotive and infrastructure [10,11,12,13,14]. The development in these sectors necessitates lightweight materials with high impact resistance and the ability to absorb significant energy upon impact. Therefore, exploring the impact performance of TI-CF FML is essential.

Earlier studies have revealed the superior mechanical properties of TI-CF FML. A study by Ali et al. [15] compared the mechanical properties and corrosion behaviour of pure titanium, CF/E composites and TI-CF FML in seawater at 70 °C after wet-heat conditioning. The results showed that the mechanical properties of TI-CF FML were slightly affected compared to CF/E composites, attributed to the water-absorbing shielding effect of the outer titanium layer. Additionally, the crevice corrosion of TI-CF FML was found to be negligible due to the formation of a stable oxide film on the titanium skin. Hu et al. [16] conducted a thermal stability test on TI-CF FML, and the results showed no delamination between the titanium layer and the composite layer even after 1000 thermal shocks, which revealed excellent thermal performance.

Given that the impact resistance, damage mechanism and energy absorption capacity of FML are crucial factors directly influencing the safe application of the material [17,18,19,20,21], TI-CF FML is required to have good impact protection performance. Studies have been undertaken to investigate the impact response of TI-CF FML. For instance, Reiner et al. [22] conducted an experimental study on the low-velocity impact response of TI-CF FML. The results concluded that composite failure was the main energy dissipation factor. Jakubczak and Bienias [23] observed that the percentage of energy absorbed by TI-CF FML increased with increasing impact energies before penetration occurs, ranging from approximately 50% at 2.5 J to about 80% at 30 J. Jakubczak [24] found that TI-CF FML exhibited strong impact resistance at low-velocity impacts of 2.5–30 J. Numerical simulation methods have proven to be effective in exploring the impact behaviour of composite laminates [25,26,27,28]. Nassier et al. [29] utilised finite element analysis techniques to model the perforation response of the investigated FML. The results provided reasonable predictions of maximum force and displacement, effectively identifying the correct failure modes within the FML specimen.

Although the prevailing research has demonstrated good impact performance of TI-CF FML, the impact responses of TI-CF FML under varying impact energies, leading from non-penetration to complete penetration of the material, have not been fully described in the literature. As a result, the energy absorption characteristics of the laminate have not been comprehensively analysed. In addition, there is a lack of research identifying the complete damage modes of the proposed laminate. Further studies on the peak force, which is a critical parameter for characterising the impact resistance of the laminate, still need to be explored.

This research aimed to thoroughly investigate the impact response and energy absorption characteristics of TI-CF FML. Experimental tests were carried out using a drop-weight impact test setup, covering a range of energy levels from 16.9 J to 91.9 J. Numerical simulations were performed in order to reveal the progressive damage of the TI-CF FML. The impact damage mechanisms, energy absorption characteristics, impact force-displacement curves, and peak force were then discussed and analysed, providing a comprehensive understanding of the low-velocity impact behaviour of TI-CF FML.

## 2. Experiment

### 2.1. Specimen

**Figure 1:**
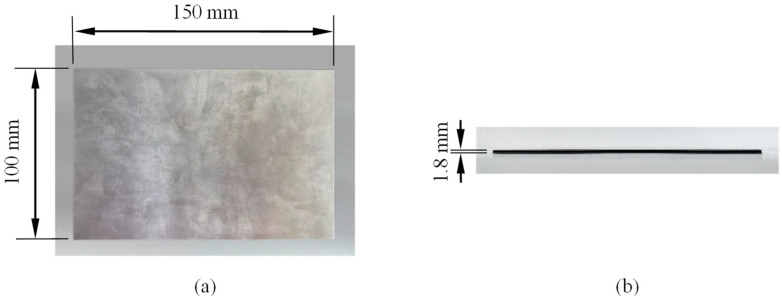
Specimen: (**a**) front view, (**b**) side view.

The TI-CF FML analysed in this study is composed of T300 carbon-fibre/epoxy prepreg (0°/90°) manufactured by Dongguan Airbus Composites Technology Co., Ltd. (Dongguan, China), with a fibre mass fraction of 58.0% and a ply thickness of 0.4 mm, interspersed with thin Ti-6Al-4V titanium alloy sheets, each with a thickness of 0.5 mm. The manufacturing process began with mechanical grit sanding surface treatment using 180-grit sandpaper on the Ti-6Al-4V sheets to increase surface roughness and enhance bonding at the metal-composite interface. A straightforward lay-up process was followed according to the lay-up configuration of Ti/(0°/90°)_2_/Ti. The assembled laminates were then vacuum-bagged and cured in an autoclave at 80 °C for two hours. A vacuum pressure of 0.09 MPa was applied throughout the curing process. The specimen was cut to dimensions of 150 mm × 100 mm, as shown in Figure 1, with a total thickness of 1.8 mm and a weight of 101.4 g.

### 2.2. Experimental Set-Up

**Figure 2:**
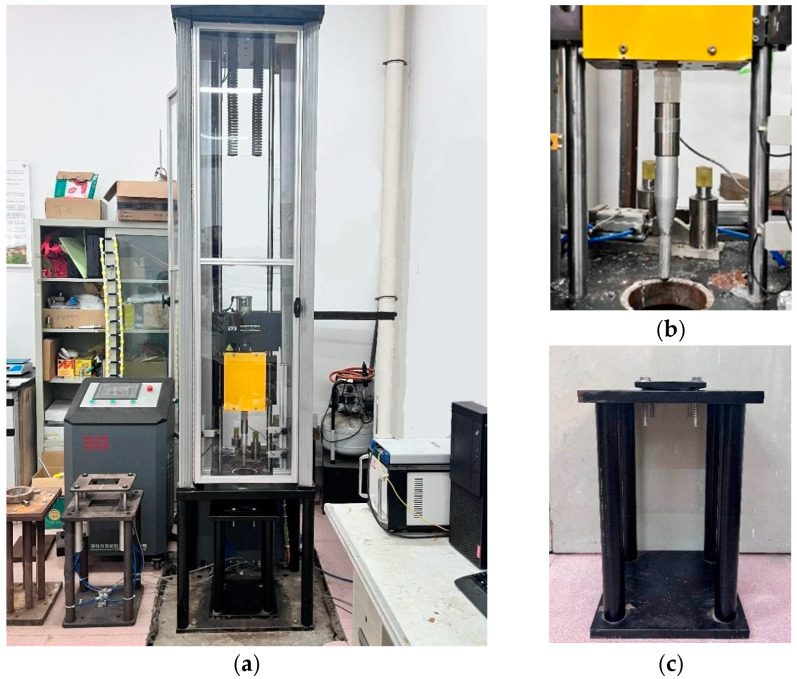
Experimental setup: (**a**) drop-weight impact testing machine, (**b**) impactor, (**c**) support fixture.

The low-velocity impact tests were carried out by the Instron Dynatup 9250 drop-weight impact tester, as shown in Figure 2a. The cylindrical impactor had a mass of 18.3 kg and a tip diameter of 14 mm (Figure 2b). The impactor struck the centre of the specimen and was released from different heights to deliver various impact energies: 16.9 J, 27.1 J, 37.0 J, 45.9 J, 53.1 J, 64.7 J, 72.8 J, 83.9 J and 91.9 J. During the impact experiment, the specimen was placed between two steel plates with an exposed area of 125 mm × 75 mm. The assembly was fixed to the supporting frame, as shown in Figure 2c, ensuring fully clamped conditions on all four sides. The impact force time-history, *F*(*t*), was recorded using a load cell. The velocity and displacement time-histories of the impactor, *v*(*t*) and *δ*(*t*), respectively, can be obtained by integrating the impact force time-history once and twice, as shown in Equations (1) and (2).

(1) $$v\left(t\right)=v_{0}-\int_{0}^{t}\frac{F\left(t\right)}{m}dt$$

(2) $$\delta \left(t\right)=\int_{0}^{t}\left(v_{0}-\int_{0}^{t}\frac{F\left(t\right)}{m}dt\right)dt$$

where *v*_0_ is the initial impact velocity in m/s, *g* is the gravity acceleration in m/s^2^, *t* is the time in s, *m* is the mass of the impactor in kg.

## 3. Numerical Analysis

### 3.1. Numerical Model

**Figure 3:**
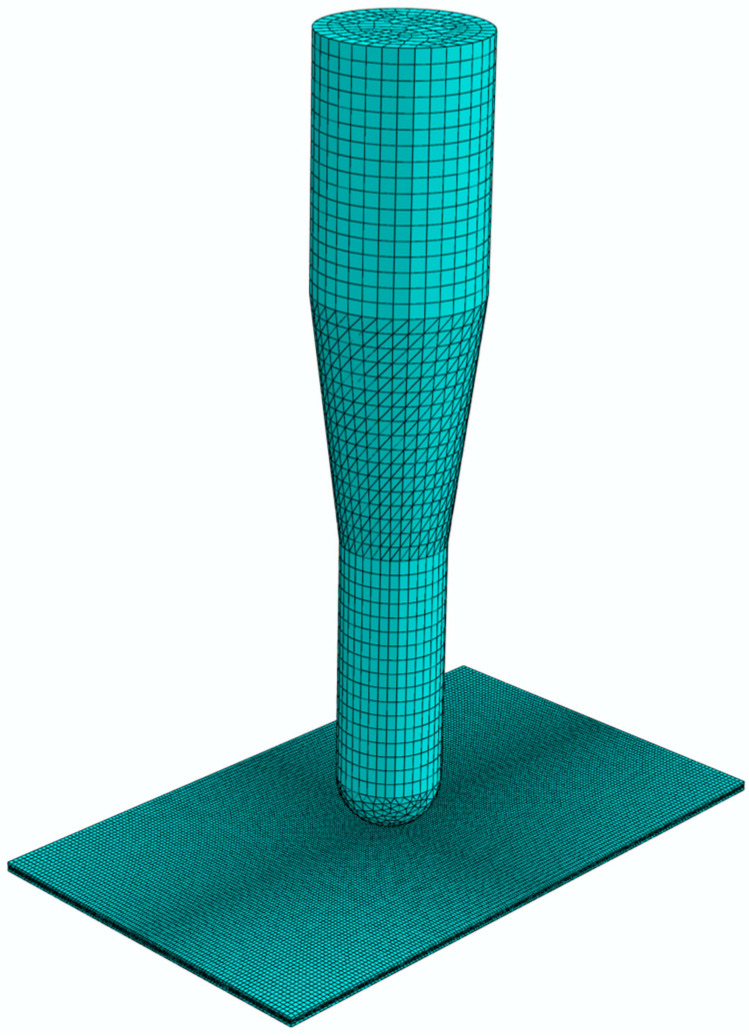
Finite element model.

The finite element analysis was completed in ABAQUS. Figure 3 displays the finite element model, consisting of a composite laminate and a falling impactor. Each layer of the composite laminate was modelled using C3D8R elements. Due to the directional nature of the laminate layers, the material orientation for each layer was defined to specify the fibre direction. The specific layup configuration was (0°/90°)_2_. A 3D zero-thickness interface element (cohesive element) was used to connect the layers. The element type for the impactor is C3D8R and is set as a rigid body. Fixed constraints were applied around the laminate to simulate the fixity of the clamping device. The finite element model constrained all degrees of freedom of the impactor except for the vertical direction. The outer surface of the impactor is exactly in contact with the centre of the surface of the laminate to ensure that the impactor remains perpendicular to the laminate during movement. The material properties of the TI-CF FML used in the finite element simulation were either provided by the supplier or obtained from published literature [30] and are summarised in Table 1. A cohesive contact model is used to describe the contact between the carbon fibre layer and the titanium layer. A friction coefficient of 0.3 is set between the impactor and the laminate. The analysis adopted a dynamic explicit calculation method, which can accurately simulate the contact and deformation during the impact process.

**Figure 4:**
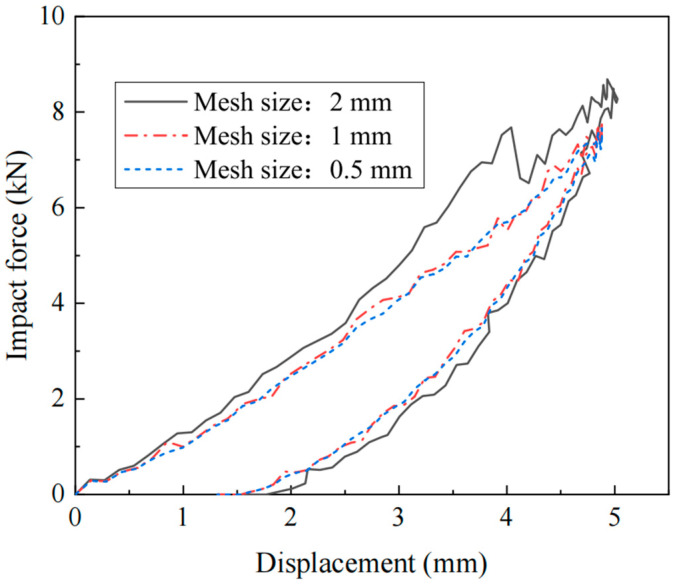
Mesh size effect.

Three mesh sizes were selected for the laminate for sensitivity analysis, specifically 2 mm, 1 mm, and 0.5 mm. Additionally, eight meshes are set along the thickness direction. Figure 4 shows the impact force and displacement curves for different mesh sizes when the impact energy is 16.9 J. It can be observed that the curves for mesh sizes of 1 mm and 0.5 mm largely overlap. Considering computational accuracy and efficiency, a mesh size of 1 mm was chosen for the simulation. During the impact, the deformation and damage of the laminate mainly occur in the central region. Therefore, a finer mesh is used in the central impact area when partitioning the mesh. There are 180 meshes along the long edge of the laminate and 110 meshes along the short edge. The mesh density gradually decreases outward from the impact point, ensuring both the accuracy of the analysis results and improved computational efficiency.

### 3.2. Numerical Results

**Figure 5:**
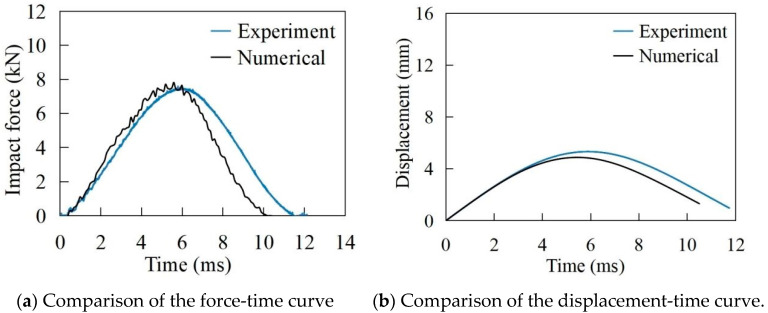
The impact energy of 16.9 J.

**Figure 6:**
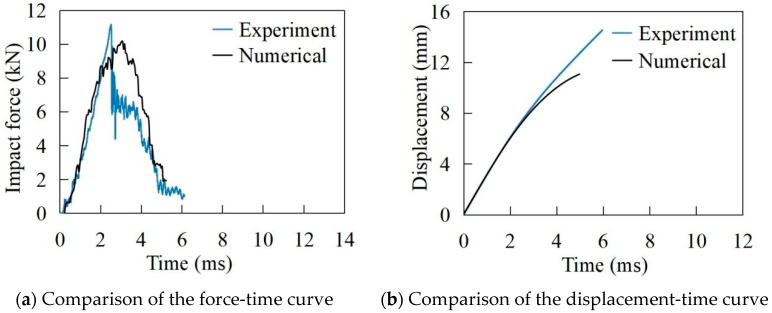
The impact energy of 91.9 J.

Figure 5 and Figure 6 show the time history of the impact force and displacement of the laminate in 16.9 J and 91.9 J impact scenarios, respectively. It is worth noting that the simulated impact force, directly extracted from ABAQUS, was calculated by multiplying the stress distribution by the contact area in the software. During the initial stage of failure, the main damage to the laminate includes interlayer delamination and matrix tension failure. The interlayer delamination and matrix failure affect the load-bearing capacity of the laminate, resulting in fluctuations in the impact load curve, and hence the oscillatory upward trend in the curve occurs. As shown in Figure 5, when the impact energy is 16.9 J, the maximum experimental value and simulated value of the impact force are 7.6 kN and 7.8 kN, respectively. The maximum displacement of the impactor is 5.3 mm and 4.9 mm, respectively. The experimental value and simulated value of the impact response time are 11.6 ms and 10.4 ms. At around 6 ms, the impact force reaches its maximum value, at which point cracks are formed in each layer due to fibre fracture. The bearing capacity of the damaged CFRP laminate is significantly reduced, leading to a decrease in the load-time curve. It can be observed from the displacement-time curve that the impactor reaches its lowest point at about 6 ms. The kinetic energy of the impactor is depleted, and the impactor starts to rebound. Afterwards, the impactor separates from the laminate, and the amplitude of the load-time curve decreases. At around 11 ms, the impactor completely separates from the laminate, and the impact load decreases to zero, indicating that the impact process is finished.

For the impact energy of 91.9 J in Figure 6, the maximum experimental and simulated values of the impact force are 11.2 kN and 10.1 kN, respectively. The maximum displacements of the impactor are 14.2 mm and 11.1 mm, and the impact response times are 5.9 ms and 5.1 ms, respectively. The maximum value of the impact force is reached at around 2.4 ms. As the destruction occurs simultaneously during this stage, the range of interlayer delamination damage and the range of matrix tensile fracture continue to expand. Eventually, a hole forms in the middle, signalling the end of the damage.

**Figure 7:**
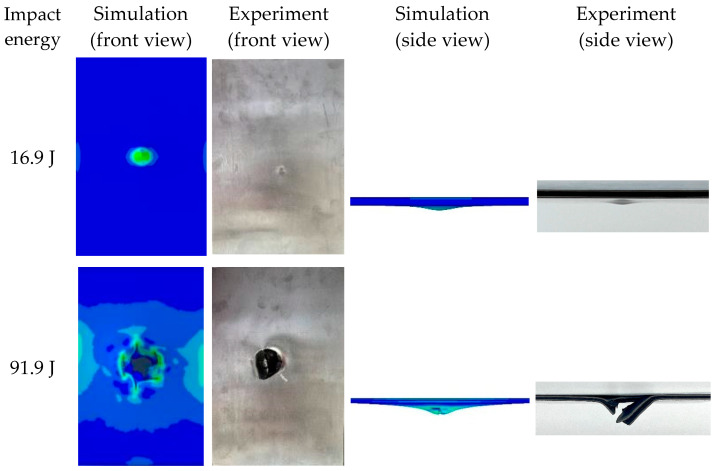
Comparison of the failure mode of the laminate.

As can be seen from Figure 5 and Figure 6, the greater the impact energy, the greater the maximum impact force and maximum displacement of the laminate. The error of the maximum impact force of finite element analysis and experiment is less than 10%. For the impact energy of 16.9 J and 91.9 J, the comparisons of the experimental and numerical failure modes of the laminate are shown in Figure 7. From Figure 5, Figure 6 and Figure 7, it can be seen that the finite element analyses are in good agreement with the test results.

## 4. Results and Discussion

### 4.1. Damage Mode

**Figure 8:**
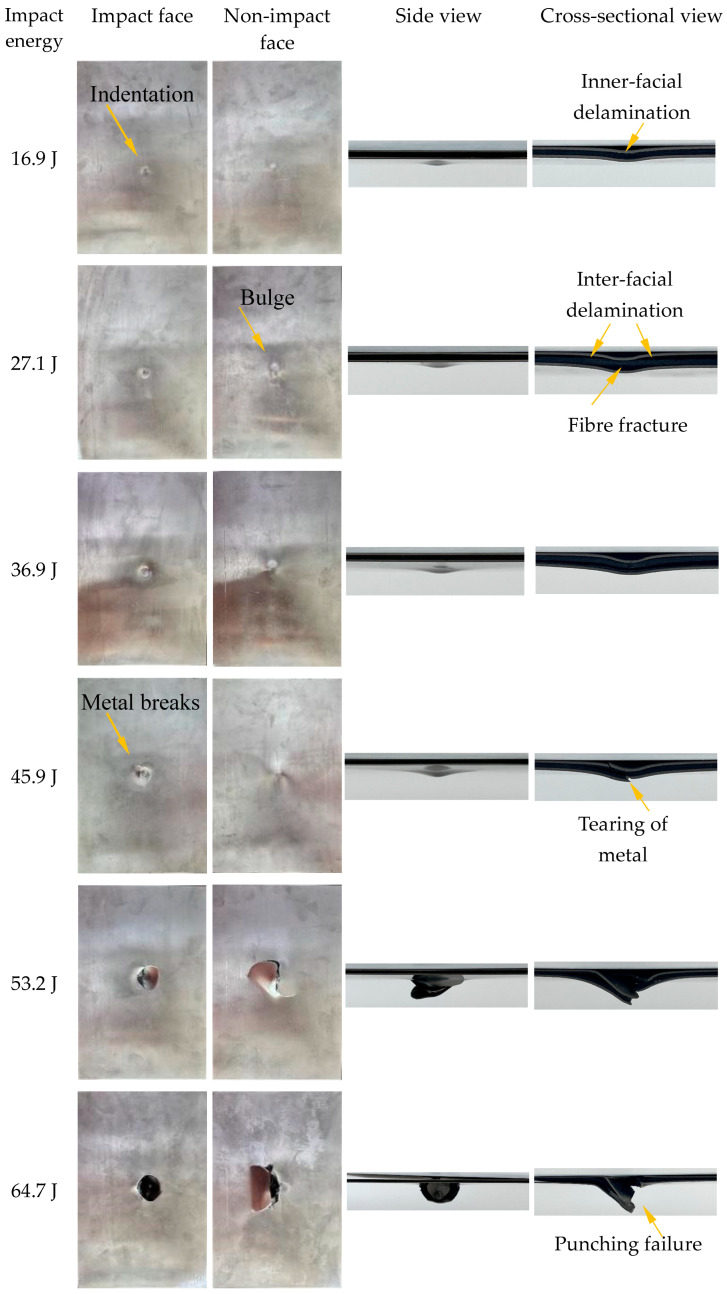

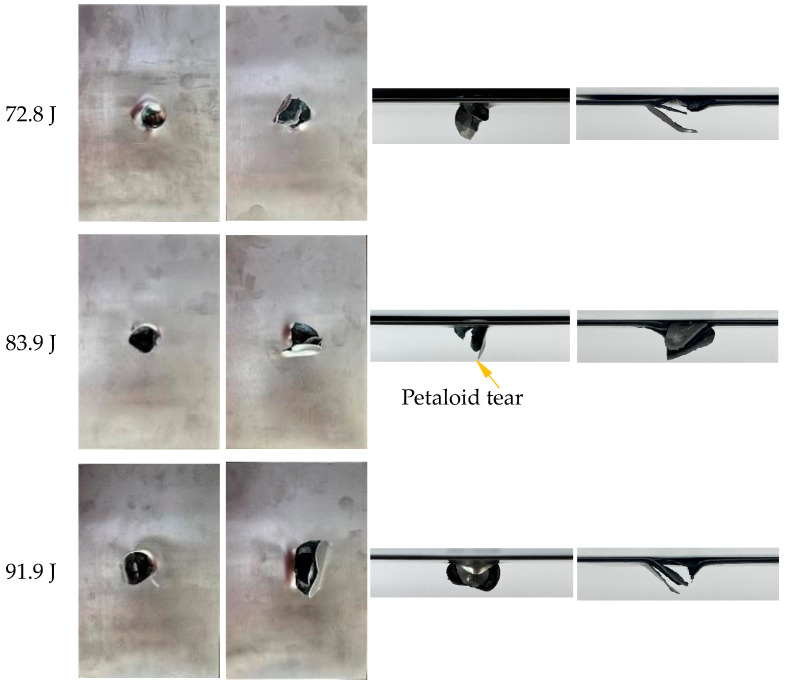
Characteristics of the impacted specimens at impact energies from 16.9 J to 91.9 J.

The impact failure mechanisms of the TI-CF FMLs are analysed in this section. The photographs of the specimens after the impact tests are depicted in Figure 8. The specimen failure mechanism can be considered as three distinct modes, which are categorised based on the observed damage behaviour under different impact energy levels.

Damage Mode I occurs for impact energies from 16.9 J to 36.9 J. In this stage, the failure mechanism involves local bulging, fibre fracture, matrix cracking as well as inner- and inter-facial delamination. As depicted in Figure 8, at an impact energy of 16.9 J, local indentation is visible on the impact side. With increasing impact energy, such as 27.1 J, local bulging starts to be observed on the non-impact side. In addition, inter-facial delamination can be seen from the cross-sectional view in Figure 8. The extent of local indentation in Mode I increases with rising impact energy. Mode II encompasses impact energies ranging from 45.9 J to 53.2 J. The metal layers in this stage begin to break on the impact side, whereas they tear on the non-impact side. Mode III signifies the punching failure of the specimen, corresponding to impact energies exceeding 64.7 J. In this damage mode, the carbon fibres completely fracture in the central impact area of the specimen, while the metal layer exhibits petaloid tearing, characterised by deformation and cracks radiating in multiple directions, forming petal-like structures. It is noteworthy that the penetration energy of 64.7 J for the TI-CF FML is more than twice that of the titanium-based glass-fibre laminate (30 J) with a similar areal density, as studied by Jakubczak [24], demonstrating the superior impact resistance of the TI-CF FML.

### 4.2. Energy Absorption

**Figure 9:**
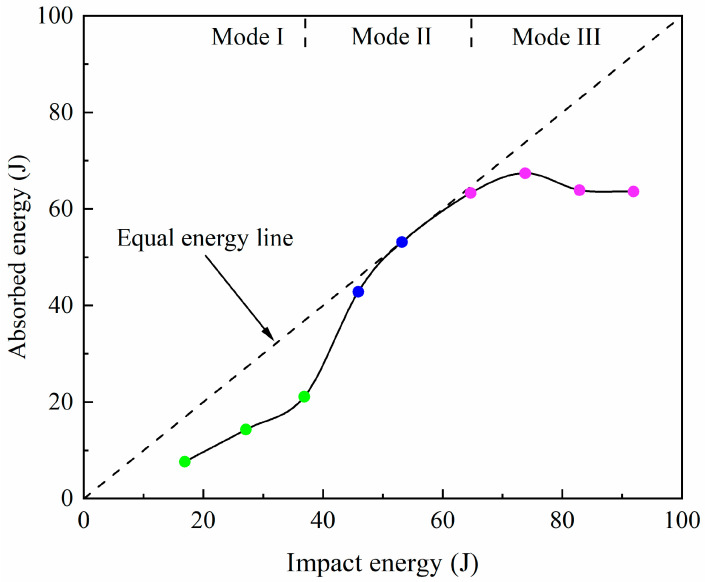
Impact energy versus absorbed energy.

The energy absorption of the laminate, a crucial parameter in evaluating the material’s resistance to damage under impact loads, is determined by the area under the impact load versus displacement curve. The impact energies and the specimen absorbed energies are listed in Table 2 and illustrated in Figure 9. The curve depicting impact energy versus absorbed energy can be divided into three regions corresponding to Mode I, Mode II and Mode III, respectively. The equal energy line, showing complete absorption of the impact energy, is plotted as a dashed line for comparison purposes in Figure 9.

In Mode I, it is evident that the absorbed energy rises with the growing impact energy. This is attributed to the increased extent of fibre fracture along with local indentation. As energy impact rises from 36.9 J to 45.9 J, there is a sharp increase in the absorbed energy, i.e., from 21.1 J to 42.8 J. This is due to the initiation of tearing in the metal layers of the specimen, leading to a transition in the damage mode to Mode II, consequently causing a surge in absorbed energy. When the impact energy reaches 53.2 J, the absorbed energy closely matches the impact energy. This indicates that the impactor becomes trapped with the specimen when passing through, and nearly the entire impact energy is absorbed by the specimen. In Mode III, the absorbed energy no longer results in a notable alteration. Complete penetration occurs, and the damage mode stays unchanged. In contrast to Jakubczak’s research [24], where titanium-based glass-fibre laminate absorbed a maximum impact energy of 23.7 J during low-velocity impact tests, the TI-CF FML in this study absorbed up to 67.4 J, demonstrating improved energy absorption characteristics.

### 4.3. Impact Force Versus Displacement Curve

**Figure 10:**
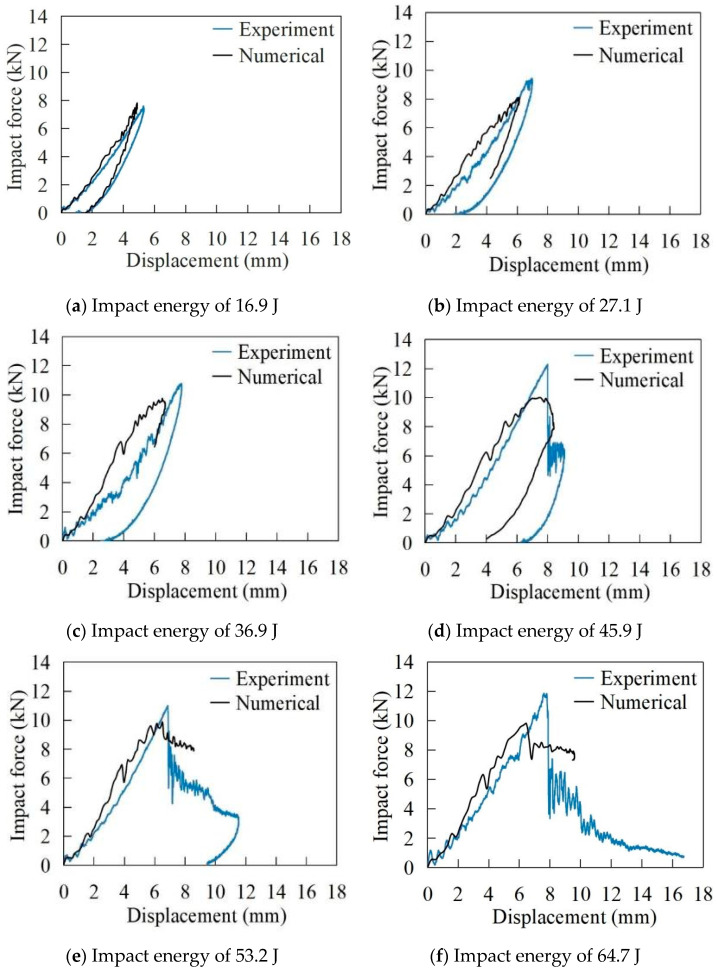

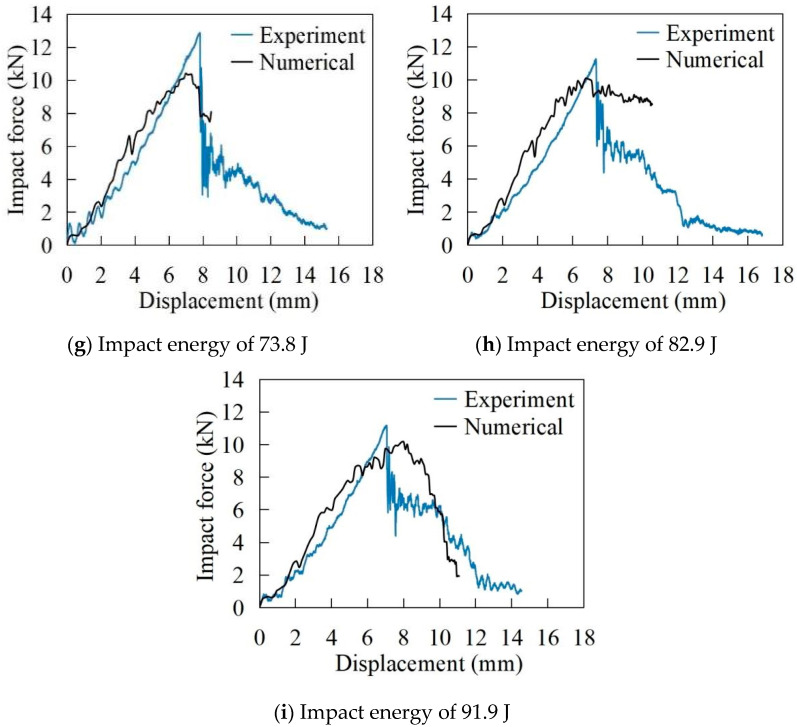
Impact force versus displacement curves at impact energies ranging from 16.9 J to 91.9 J.

Figure 10 displays the numerical and experimental results of the impact force versus displacement curves of the specimen for impact energies ranging from 16.9 J to 91.9 J. It is evident that the simulated curve aligns well with the experimental curve.

In Figure 10a–c, the impact forces rise as displacement increases until reaching their peak values. Subsequently, the impact forces begin to decrease as the displacement reduces, indicating the rebounding of the impactor. The final displacement will not return to zero, and its value is equal to the localised indentation of the specimen. In Figure 10d,e, it can be seen that after the impact forces reach their peak values, the curves exhibit a sudden drop, which is attributed to the breakage of the metal skin. Then, the curves begin to oscillate before the displacement starts to decrease. This behaviour is a result of inner- and inter-facial delamination, fibre breakage as well as matrix cracking. The impactor may either bounce back or become trapped in the laminate, and the specimen either not or partially penetrates. However, in Figure 10f–i, the displacement of the impactor does not decrease throughout the entire impact process, indicating a complete penetration of the laminate.

### 4.4. Peak Force Versus Impact Energy Curve

**Figure 11:**
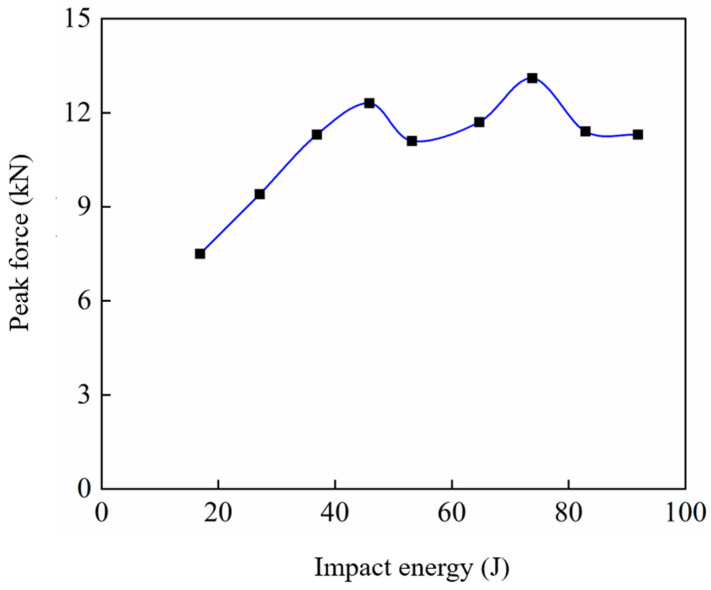
Peak force versus impact energy.

The experimentally measured peak force and impact energy are listed in Table 3 and illustrated in Figure 11. For impact energies ranging from 16.9 J to 36.9 J (Mode I), the peak force rises with increasing impact energy before stabilising. Subsequently, the peak force reaches a plateau, fluctuating between 11.1 kN and 13.1 kN. Notably, at an impact energy of 73.8 J, the force peaks at 13.1 kN, which is more than twice the 5.5 kN measured for the titanium-based glass-fibre laminate in the published research [24]. The specimen’s penetration energy falls within this range, representing the maximum force that the specimen can endure.

## 5. Conclusions

In this paper, the low-velocity impact behaviour of TI-CF FML was examined through experimental and numerical simulations. The impact failure mechanisms of TI-CF FML were categorised into three distinct damage modes based on the impact energies: Mode I features local bulging, Mode II exhibits localised indentation accompanied by metal breakage, whereas Mode III characterises punching failure. Fibre fracture, matrix cracking, as well as inner- and inter-facial delamination are involved in three damage modes. The absorbed energy increases with rising impact energy for non-penetrating scenarios in Modes I and II; however, it remains constant when penetration occurs in Mode III. The numerically simulated impact force versus displacement curves aligned well with experimental findings, showing that the laminate can withstand a peak force of 13.1 kN. The energy absorption capability and the peak force of the TI-CF FML are both more than twice those exhibited by the titanium-based glass-fibre laminate with the same area density. The results obtained from this research revealed the low-velocity impact response of TI-CF FML, providing an in-depth understanding of the laminate’s performance under unexpected impact loads. This study is fundamentally important towards real industrial applications in future research and design.

## Figures and Tables

**Table 1:** Material properties of the TI-CF FML.

Material	Constants	Value	Constants	Value	Constants	Value
Titanium layers	*E* (GPa)	113.5	*A* (MPa)	931		
	*v*	0.3	*ρ* (kg/m^3^)	5870		
Fibre composite layers	*E_f_*_1_ (GPa)	130	*G_f_*_12_ (GPa)	3.6	*X*_T_ (MPa)	1760
	*E_f_*_2_ (GPa)	7.1	*G_f_*_13_ (GPa)	3.6	*X*_C_ (MPa)	1100
	*E_f_*_3_ (GPa)	7.1	*G_f_*_23_ (GPa)	3.08	*Y*_T_ (MPa)	51
	*v*_12_	0.32	*S*_12_ (MPa)	70	*Y*_C_ (MPa)	167
	*v*_13_	0.32	*S*_13_ (MPa)	70	*S* (MPa)	45
	*v*_23_	0.52	*S*_23_ (MPa)	70	*ρ* (kg/m^3^)	1690
Cohesive layers	*N* (MPa)	60	*G*_I_ (MPa)	0.35	*k* (MPa/mm)	10^6^
	*S* (MPa)	80	*G*_II_ (MPa)	1.45	*ρ* (kg/m^3^)	1480
	*T* (MPa)	80	*G*_III_ (MPa)	1.45		

where 1, 2, and 3 indicate the direction. *ρ* is the density; *E* is the Young’s modulus; *v* is the Poisson’s ratio; *A* is the yield stress; *X*_T_, *X*_C_, *Y*_T_, and *Y*_C_ are axial tensile, axial compression, transverse tensile, and transverse compressive strengths, respectively; *S*_12_, *S*_13_ and *S*_23_ are the shear strengths; *k* is the initial stiffness; *N*, *S* and *T* are the tensile and shear strengths of interface, respectively. *G*_I_, *G*_II_ and *G*_III_ are the current energy release rates.

**Table 2:** Impact energy and absorbed energy.

Impact Energy (J)	Absorbed Energy (J)
16.9	7.6
27.1	14.3
36.9	21.1
45.9	42.8
53.2	53.1
64.7	63.3
73.8	67.4
82.9	63.9
91.9	63.6

**Table 3:** Impact energy and peak force.

Impact Energy (J)	Peak Force (kN)
16.9	7.5
27.1	9.4
36.9	11.3
45.9	12.3
53.2	11.1
64.7	11.7
73.8	13.1
82.9	11.4
91.9	11.3

## Data Availability

Data is available upon request.
